# Access to molecular diagnostics for CNS tumors through international outsourcing: experience from Jordan

**DOI:** 10.3389/fonc.2026.1855266

**Published:** 2026-07-17

**Authors:** Ruba Al Abweh, Sarah Al Sharie, Nabil Hasasna, Nisreen Amayiri, Mouness Obeidat, Maysa Al-Hussaini

**Affiliations:** 1 Department of Cell Therapy and Applied Genomics. King Hussein Cancer Center, Amman, Jordan; 2 Department of Pathology and Immunology, Washington University in St. Louis, St. Louis, MO, United States; 3 Department of Pediatric Oncology, King Hussein Cancer Center, Amman, Jordan; 4 Department of Surgery, Unit of Neurosurgery, King Hussein Cancer Center, Amman, Jordan

**Keywords:** CNS tumors, LMIC (low and middle income countries), molecular diagnosis, out-sourcing, targeted therapy

## Abstract

**Background:**

Molecular profiling is now integral to the diagnosis, risk stratification, and treatment of central nervous system (CNS) tumors following the 2021 WHO Classification (WHO CNS5). However, access to molecular diagnostics remains severely limited in settings with limited resources. International outsourcing to accredited reference laboratories represents a potential bridging strategy, yet systematic data on its feasibility and clinical impact in the LMICs are lacking.

**Methods:**

We conducted a retrospective review of CNS tumor cases at King Hussein Cancer Center (KHCC), Amman, Jordan, that underwent outsourced molecular testing at the Hospital for Sick Children in Toronto, Canada, between 2021 and 2023. Four test types were ordered: medulloblastoma subgrouping by NanoString nCounter-based gene expression profiling, TruSight pan-cancer RNA sequencing, low-grade fusion gene analysis, and C19MC fluorescence *in situ* hybridization (FISH). For each case, we recorded turnaround time (TAT), cost, conclusive result rate, impact on diagnosis, and identification of actionable therapeutic targets.

**Results:**

A total of 105 patients underwent outsourced molecular tumor testing (87% pediatric; median age 10 years; 54% male). Tumor types included medulloblastoma (52%), low-grade glioma (LGG, 30%), high-grade glioma (HGG, 12%), and others (6%). Of the 119 samples that reached the reference laboratory, 109 (92%) yielded a conclusive molecular result, with the highest rate in medulloblastoma (94%) and the lowest in ependymoma (33%). The median overall TAT was 26 days (range 13–140 days), and the total expenditure was 153,506 US Dollars. Molecular testing led to a change in diagnosis in 4 cases (4%): 3 major changes (reclassification of BCOR sarcoma to solitary fibrous tumor, ependymoma to pilocytic astrocytoma, and pediatric-type diffuse low-grade glioma with *COL1A1::PDGFB* fusion) and 1 minor change. In one additional case, a discordant molecular result (*NRAS*-mutant low-grade glioma) did not alter the final morphological diagnosis of high-grade glioma. Actionable targets (predominantly *BRAF* alterations) were identified in 28 tumors (27%), including 65% of LGGs and 54% of HGGs.

**Conclusion:**

International outsourcing of molecular diagnostics is a feasible and clinically impactful strategy for CNS tumor management at limited-resource settings, enabling WHO CNS5-integrated diagnoses and access to precision therapy. The TAT and cost per tumor sample are relatively acceptable given the benefits of a more accurate diagnosis and the possibility of finding a targetable alteration. Permanent solutions would require investment in regional molecular diagnostic infrastructure through in-house capacity building, laboratory networks, and international twinning programs.

## Introduction

Central nervous system (CNS) tumors represent a heterogeneous group of neoplasms with markedly variable biological behavior, prognosis, and treatment response (1). Among pediatric malignancies, CNS tumors are the most common solid tumors and the leading cause of cancer-related mortality, underscoring the critical importance of accurate diagnosis and timely initiation of appropriate therapy (2). Over the past two decades, advances in molecular pathology have fundamentally transformed the classification and management of CNS tumors (3–6). The 2021 World Health Organization (WHO) Classification of Tumors of the Central Nervous System (CNS5) emphasized an integrated diagnostic framework that incorporates molecular markers alongside histological findings, establishing molecular profiling as essential rather than optional for defining tumor type, grade, and prognosis (3).

Molecular testing has become particularly pivotal in several CNS tumor subtypes (7). In medulloblastoma, subgroup classification into WNT-activated, SHH-activated, and non-WNT/non-SHH molecular groups carries direct prognostic and therapeutic implications, enabling risk stratification that may inform treatment decisions in protocol-based settings (8). In low-grade gliomas, identification of *BRAF* fusions, BRAF p.V600E mutations, and other targetable alterations has enabled investigational molecularly targeted therapies, including BRAF and MEK inhibitors, which have shown efficacy in clinical trials and are increasingly available through compassionate access programs (9, 10). In high-grade gliomas, *IDH* mutation status, and *EGFR* amplification, are now integral to diagnosis and prognostic assessment (11). Similarly, molecular confirmation of C19MC amplification is required for the diagnosis of embryonal tumor with multilayered rosettes (ETMR), and *RELA* or *YAP1* fusions define clinically relevant ependymoma subtypes (12).

Despite the centrality of molecular diagnostics to modern neuro-oncology, access to these tests remains profoundly unequal across healthcare systems (13). In better resourced settings, advanced molecular diagnostics, including RNA fusion panels, and DNA methylation profiling, are increasingly available, though often centralized in specialized academic centers or integrated through collaborative networks rather than universally accessible in routine practice (14). In contrast, many centers in low- and middle-income countries (LMICs), including those in the Middle East and North Africa (MENA) region, face substantial barriers to molecular testing, including limited laboratory infrastructure, lack of certified pathologists with molecular expertise, prohibitive costs, and absence of locally validated testing platforms (15, 16). These disparities translate directly into diagnostic and therapeutic inequities, with patients in resource-limited settings receiving diagnoses that are incomplete by contemporary standards, and consequently missing opportunities for targeted therapy or appropriate risk-adapted treatment (17).

This study describes the two-year experience at KHCC with outsourcing molecular testing for CNS tumors between 2021 and 2023. We evaluated the feasibility and clinical utility of this approach.

## Methods

We conducted a retrospective study of adult and pediatric CNS tumor cases referred for molecular testing from KHCC in Jordan to The Hospital for Sick Children (SickKids) in Canada between November 2021 and December 2023. At KHCC, the multi-disciplinary clinic (MDC) agreed on 115 patients with confirmed pathology diagnoses for further molecular stratification by the following tests provided by the SickKids laboratory; Medulloblastoma subgrouping by NanoString, Tru-Sight pan-cancer RNA sequencing, low-grade fusion analysis, and C19MC by fluorescence *in situ* hybridization (FISH).

KHCC operates as a comprehensive cancer center within a limited-resource settings. Using the NCCN Framework for Resource Stratification of Cancer Treatment Recommendations, KHCC functions at an enhanced resource level, defined by availability of pathology services including immunohistochemistry and limited molecular diagnostics, access to standard chemotherapy and radiation therapy, and sporadic access to targeted therapies primarily through compassionate use programs rather than routine formulary availability or clinical trial enrollment. During the study period (2021-2023), in-house molecular diagnostic capabilities were limited to basic immunohistochemistry and fluorescence *in situ* hybridization for select targets, with no available platforms for RNA sequencing, DNA methylation profiling, or comprehensive next-generation sequencing panels (16). Targeted therapy access was restricted to BRAF and MEK inhibitors (dabrafenib, trametinib) obtainable through pharmaceutical compassionate access programs for select patients with BRAF-altered tumors; broader targeted agents such as IDH inhibitors, NTRK inhibitors, or investigational therapies remained largely inaccessible outside of exceptional individual requests. This resource profile directly informed the selection of molecular tests for outsourcing, prioritizing those with the highest likelihood of identifying alterations for which therapeutic access, albeit limited, was established or potentially attainable.

SickKids performed molecular testing only; no central pathology review, slide re-review, or immunohistochemistry reinterpretation was conducted as part of the outsourcing service. All morphological diagnoses and immunohistochemical interpretations were performed locally at KHCC by a board-certified neuropathologist. The integrated final diagnoses incorporating molecular findings were rendered collaboratively through discussion between KHCC pathologists and the referring clinicians during MDC meetings, considering the local histopathological diagnosis, immunohistochemical profile, and the molecular results returned from SickKids. In cases where molecular findings were discordant with the initial morphological diagnosis, slides were re-reviewed locally at KHCC, and the final integrated diagnosis was established according to WHO CNS5 criteria. In a few instances, the findings were discussed during the monthly joint meeting between KHCC and SickKids group under the umbrella of the twinning program (18).

Formalin-fixed paraffin-embedded (FFPE) tissue blocks were shipped from KHCC to SickKids via international courier service. During the study period, samples were lost during shipment, prompting implementation of additional safeguards, including photographic documentation of packaged specimens, retention of backup blocks when tissue quantity permitted, and enhanced communication protocols with the courier service.

The existing twinning partnership between KHCC and SickKids facilitated access to molecular testing at standard institutional rates without special discounts or research collaboration agreements; testing was performed on a fee-for-service basis at SickKids’ published institutional pricing for external referring centers.

The four specific tests were selected based on their anticipated impact on clinical management within our resource-limited setting. Medulloblastoma subgrouping by NanoString was prioritized because molecular subgroup assignment (WNT, SHH, Group 3, Group 4) is mandated by contemporary treatment protocols and directly influences treatment intensity, particularly the addition of carboplatin to radiotherapy for high-risk Group 3 tumors, a decision that cannot be made on histological grounds alone. The TruSight pan-cancer RNA sequencing panel was selected for its broad utility in identifying actionable fusion genes across multiple tumor types, most importantly *BRAF* fusions and BRAF p.V600E mutations in low-grade and high-grade gliomas, given our institutional access to BRAF and MEK inhibitors through compassionate use programs; this test also enabled the detection of other targetable alterations such as *NTRK*, *RET*, and *ALK* fusions. Low-grade fusion gene analysis was employed as a focused, cost-effective alternative to the broader TruSight panel when clinical suspicion was high for a specific *BRAF*-altered low-grade glioma and financial constraints precluded the more expensive comprehensive panel. C19MC FISH was utilized for diagnostically ambiguous embryonal tumors with rosette-forming features, as C19MC amplification is required for the diagnosis of embryonal tumor with multilayered rosettes (ETMR) and carries distinct prognostic implications. Collectively, these tests were chosen to maximize clinical utility by addressing the most common actionable alterations and diagnostically essential molecular markers relevant to CNS tumor management in our patient population, while balancing cost considerations inherent to a limited-resource setting.

Case selection for outsourced molecular testing was determined during the weekly neuro-oncology MDC meetings based on defined clinical indications. Specific indications included: (1) all medulloblastoma cases, for which molecular subgrouping was mandated per institutional clinical practice guidelines to guide risk-stratified therapy; (2) low-grade glioma cases with progressive, recurrent, or refractory disease where *BRAF* alteration testing was indicated given available compassionate access to BRAF and MEK inhibitors; (3) diagnostically challenging cases where histopathological features were ambiguous or discordant with radiological or clinical findings, necessitating molecular confirmation; (4) high-grade glioma cases where molecular profiling could identify actionable alterations (such as *IDH* mutations, *BRAF* alterations, or other targetable drivers) or refine prognostic classification; and (5) rare embryonal tumors requiring molecular confirmation, such as C19MC amplification for ETMR. Cases were excluded from outsourcing when the institutional pathologist deemed the tissue quantity insufficient, when the diagnosis was straightforward and molecular testing would not alter clinical management, or when financial constraints precluded testing despite a clinical indication.

We examined patient demographics along with a range of clinicopathological characteristics. Additional parameters analyzed included the average turnaround time (TAT) in working days and total cost in Jordanian dinars (JD) and US Dollars. We also evaluated the implications of outsourcing on the diagnostic accuracy and management decisions of patients.

Descriptive statistical analysis, including counts and percentages, and frequencies, was performed using R Statistical Software (version 4.5.2; R Core Team, 2025). The study was approved by KHCC Institutional Review Board (IRB) number 23 KHCC 68. A waiver of informed consent was granted, as this is a minimal-risk retrospective study with no direct intervention or interaction with participants.

## Results

### Patient and tumor characteristics

Between 2021 and 2023, a total of 129 pathology samples from 115 patients with CNS tumors were considered for outsourcing. Of the 129 samples, 10 samples could not be shipped due to tissue block unavailability (n=6) or financial constraints (n=4). A total of 105 unique patients (119 samples) reached the reference laboratory and constitute the final study cohort; of these, 109 samples (92%) yielded conclusive molecular results. The cohort was predominantly pediatric, with 91 patients (87%) under 18 years of age and a median age of 10 years (range 1.5–45 years). Fifty-seven patients (54%) were male and 48 (46%) were female.

**Figure 1:**
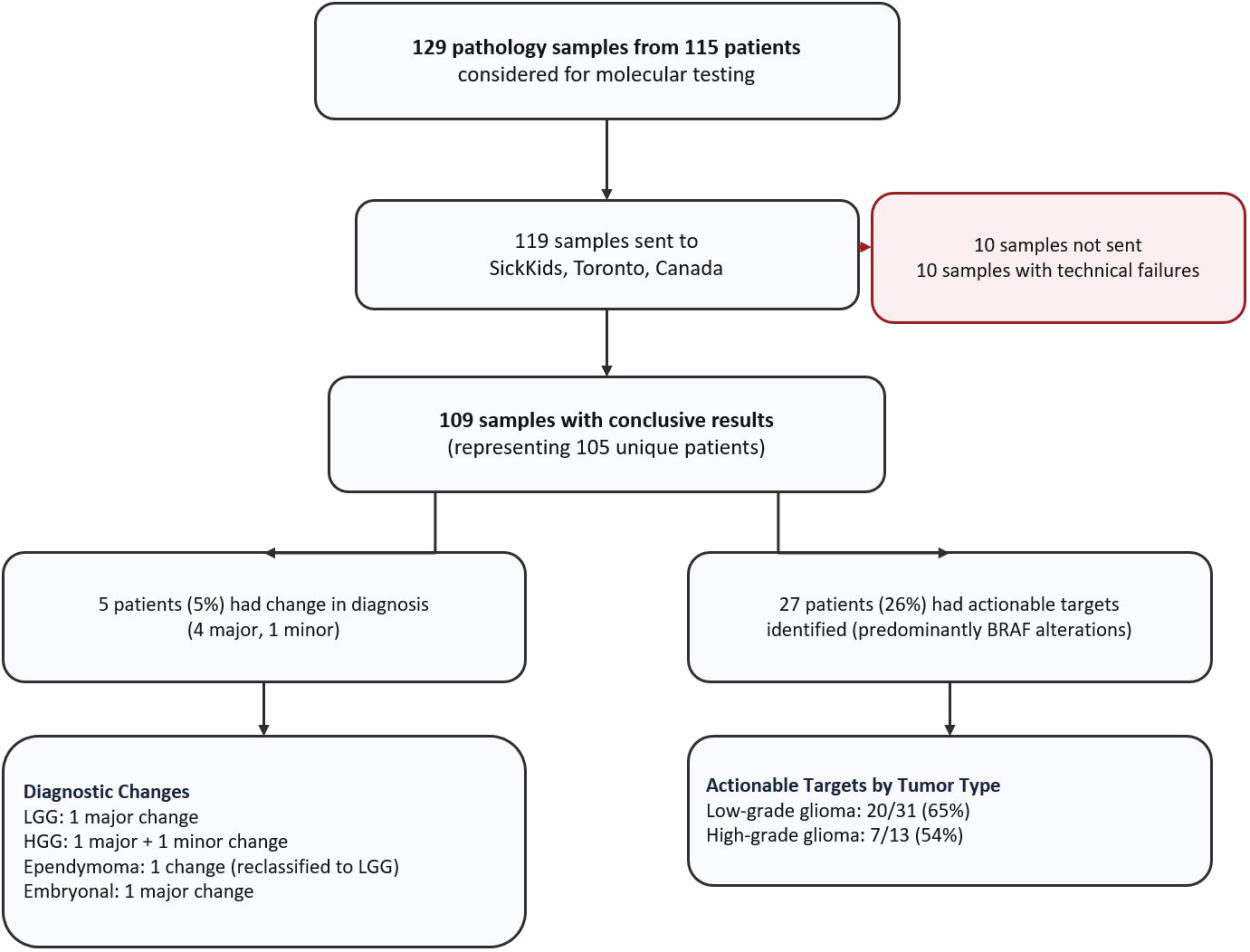
Flowchart of sample processing and clinical outcomes following outsourced molecular testing.

Notably, 12 patients had more than one sample submitted: 5 underwent two or more different tests on the same or different specimens (often in the context of disease progression), and 7 had the same test re-sent due to sample loss, inadequate tissue, or financial issues. The most common tumor diagnosis was medulloblastoma (55 patients, 52%), followed by low-grade glioma (31 patients, 30%), high-grade glioma (13 patients, 12%), embryonal tumors (4 patients, 4%), ependymoma (1 patient, 1%), and others (1 patient, 1%) (**Table 1**). The overall sample flow and clinical outcomes are summarized in **Figure 1**.

**Table 1:** Patient and tumor characteristics.

Characteristic	n	%
Age (105 patients)		
Range (1.5–45 years)		
Pediatric (<18 years)	91	87%
Adult (≥18 years)	14	13%
Gender		
Male	57	54%
Female	48	46%
Tumor Diagnosis		
Medulloblastoma	55	52%
Low-grade glioma	31	30%
High-grade glioma	13	12%
Embryonal tumor (non-MB), including 1 case of Embryonal tumor with multilayered rosettes (ETMR)	4	4%
Ependymoma	1	1%
Intracranial mesenchymal tumor	1	1%

### Molecular tests performed, turnaround time, and cost

Four categories of molecular tests were performed; among the 109 samples that yielded conclusive results, the distribution was as follows: TruSight pan-cancer RNA sequencing panel in 48 samples (44%), medulloblastoma subgrouping in 54 samples (50%), low-grade fusion gene analysis in 4 samples (4%), and C19MC fluorescence *in situ* hybridization (FISH) in 3 samples (3%) (**Table 2**).

**Table 2:** Molecular test comparing categories against turnaround time and cost.

Test type	n (%)	Median TAT, days (range)	Mean TAT, days	Cost/sample (JOD/USD)	Total cost (JOD/USD)
TruSight pan-cancer RNAseq	48 (44%)	29 (17–140)	34.6	1,652/2,327	79,296/111,696
MB subgrouping	54 (50%)	20 (13–63)	25.1	485/683	26,190/36,882
Low-grade fusion gene analysis	4 (4%)	20 (14–42)	24.2	500/704	2,000/2,816
C19MC by FISH	3 (3%)	21 (21–33)	25.0	500/704	1,500/2,112
Total	109 (100%)	26 (13–140)	29.3	—	108,986/153,506

TAT, Turnaround time, calculated from date of shipment to date of result receipt; JOD, Jordanian Dinar; USD, US Dollars; MB, Medulloblastoma subgrouping; FISH, Fluorescence *in situ* hybridization. The samples with technical failures were not charged by the reference laboratory and are excluded from this table.

The overall median turnaround time (TAT), calculated from the date of shipment to the date of result receipt, was 26 days (range 13–140 days), with a mean TAT of 29.3 days. The median TAT remained the same (26 days) after the exclusion of the sample with the longest TAT (140 days, TruSight pan-cancer RNAseq panel), due to sample loss requiring re-shipment. The median TAT was 20 days for medulloblastoma subgrouping (range 13-63) and low-grade fusion gene analysis (range 14-42), and 21 days for C19MC FISH (range 21-33), while it was 29 days for the TruSight pan-cancer RNAseq panel (range 17-140).

The cost per sample ranged from USD 683 (485 JOD) for medulloblastoma subgrouping to USD 2,327 (1,652 JOD) for the TruSight pan-cancer RNAseq panel, with low-grade fusion gene analysis and C19MC FISH each priced at USD 704 (500 JOD). The total expenditure across the study period was approximately 153,506 USD (108,986 JOD), representing costs for the 109 samples that yielded conclusive results, including the repeated samples and excluding the 10 samples that failed testing. The TruSight RNAseq panel accounted for the largest share at 111,696 USD (79,296 JOD), driven by both its higher per-sample cost and its frequent utilization.

### Molecular findings and clinical impact

Overall, 109 of 119 samples that reached SickKids (92%) yielded a definitive final molecular report, representing 105 unique patients. The rate of conclusive results varied considerably by tumor type. Medulloblastoma demonstrated the highest reporting rate at 94% (58/62 samples, representing 55 unique patients), while ependymoma had the lowest at 33% (1/3 samples, 1 unique patient). Final diagnoses were 84% (31/37 samples, 31 unique patients) for low-grade glioma, 72% (13/18 samples, 13 unique patients) for high-grade glioma, and 63% (5/8 samples, 4 unique patients) for embryonal tumors (**Table 3**).

**Table 3:** Molecular findings and clinical impact by diagnosis group.

Diagnosis group	Samples considered (n)	Conclusive results n (%)	Unique patients with results	Diagnostic change (n)	Actionable targets n (%)
Medulloblastoma	62	58 (94%)	55	0	0 (0%)
Low-grade glioma	37	31 (84%)	31	1	20 (65%)
High-grade glioma	18	13 (72%)	13	1	7 (54%)
Ependymoma	3	1 (33%)	1	1	1 (100%)
Embryonal tumor (other than MB)	8	5 (63%)	4	1	0 (0%)
Others	1	1 (100%)	1	0	0 (0%)
Total	129	109 (84%)	105	4	28 (27%)

The samples considered column reflects all 129 samples considered for outsourcing; of these, 119 reached the reference laboratory and 109 yielded conclusive results. Conclusive results were therefore obtained in 109 of 129 samples considered (84%), or 109 of 119 samples that reached the laboratory (92%). Diagnosis groups reflect the working diagnosis under which each case was submitted for testing, which may differ from the final integrated diagnosis after reclassification (**Table 1** reflects final diagnoses). The identification of actionable molecular alterations in 28 of 105 patients (27%), representing cases where therapeutically targetable alterations were detected for which matched targeted agents exist or are in clinical development, was the most significant clinical finding. This was most pronounced in low-grade glioma, where 20 of 31 patients (65%) had at least one targetable alteration identified, predominantly *BRAF* fusions or BRAF p.V600E mutations amenable to BRAF and MEK inhibitor therapy. In high-grade glioma, 7 of 13 patients (54%) had actionable findings. One of the tumors initially diagnosed as ependymoma was subsequently reclassified as low-grade glioma (pilocytic astrocytoma) following molecular analysis, for which an actionable alteration was identified. Among the 28 patients with actionable molecular alterations, the vast majority were detected using the TruSight pan-cancer RNA sequencing panel.

Molecular testing led to a change in diagnosis in 4 of 105 cases overall (4%). Among these, 3 were major changes and 1 was a minor change. Diagnostic changes were distributed across tumor types as follows: low-grade glioma and embryonal tumor each had one major diagnostic change (3% and 20% of their respective groups), high-grade glioma had one minor change (8%), and ependymoma had one change. Medulloblastoma had no diagnostic changes.

Major diagnostic changes were defined as cases in which molecular findings led to a new diagnosis that differed from the original histopathological diagnosis, resulting in a substantive change in the management plan. Minor changes were defined as alterations in tumor grade within the same diagnostic category, which did not have major implications for the treatment approach.

The first major change involved a tumor originally diagnosed as undifferentiated round cell sarcoma with *BCOR* genetic alteration, which was reclassified following identification of a *NAB2::STAT6* fusion transcript, consistent with solitary fibrous tumor. The second major change involved a supratentorial ependymoma, CNS WHO grade 2, that was reclassified as pilocytic astrocytoma following detection of a *JAKMIP2* (exon 20)::*BRAF* (exon 9) fusion. In one further case, that of a pediatric-type diffuse high-grade glioma (H3-wildtype), molecular analysis revealed *NRAS* p.Q61K and *NF1* p.V33Sfs*9 variants, suggestive of a low-grade glioma; this molecular impression was discordant with the morphology, and after local slide re-review the final integrated diagnosis remained high-grade glioma, so this case was not counted as a diagnostic change. The third major change was a pediatric-type diffuse low-grade glioma in which a *COL1A1::PDGFB* fusion was identified, altering the diagnostic classification and opening potential targeted therapeutic avenues. The single minor change involved a case originally diagnosed as diffuse hemispheric glioma, H3 G34-mutant, in which molecular profiling identified *IDH1* p.R132C, *TP53* p.M237I, *ATRX* p.K1361*, and *PIK3R1* p.N564D mutations, with findings consistent with IDH-mutant astrocytoma, representing a reclassification within the high-grade glioma category without a fundamental change in the overall management approach.

### Challenges, including technical failure

Overall, 20 of the 129 samples considered for outsourcing did not yield a final molecular diagnosis despite the MDC recommendation: 10 were never shipped (tissue block unavailable, n=6; financial constraints, n=4), and 10 of the 119 samples that reached SickKids (8%) failed to yield a conclusive result. Among these 10 technical failures, the causes were insufficient tumor tissue for molecular analysis (n=4), poor RNA quality or degradation in FFPE tissue (n=3), inadequate sequencing quality (n=2), and inability to confidently assign a molecular subgroup (n=1); failures were concentrated in RNA-based sequencing. These technical failures should be distinguished from repeat submissions, as some patients required re-submission when an initial sample failed, creating partial overlap between these categories.

## Discussion

This study describes a two-year experience at KHCC with outsourcing molecular diagnostics for CNS tumors to an internationally accredited reference laboratory, encompassing 105 patients with evaluable molecular results from 109 of 119 samples sent between November 2021 and December 2023.

The 2021 WHO Classification of CNS tumors fundamentally reshaped the diagnosis of CNS tumors from histology-only diagnoses, which are now regarded as incomplete by contemporary standards and carry the NOS (not otherwise specified) designation when molecular data are unavailable, to a more refined, molecularly integrated diagnosis (21). Specific molecular alterations now integral to diagnostic criteria include *IDH1/2* mutations, *TERT* promoter mutations, *CDKN2A/B* homozygous deletions, and *BRAF* alterations including fusions, among others (19). In this context, the inability to perform in-house molecular testing is not merely a resource limitation; it is a direct barrier to providing integrated diagnoses according to the WHO CNS5 criteria. The outsourcing program described here was established precisely to bridge this gap, and the overall 92% conclusive final reports confirm its practical effectiveness.

Access to molecular diagnostics for CNS tumors remains profoundly unequal globally. A survey across 19 countries in the Asian Oceanian region found that the availability of molecular techniques in-house was limited in low-income and lower middle-income countries, with only 29% having fluorescence *in situ* hybridization, 10.7% Sanger sequencing, and only 9.4% next-generation sequencing (20). Outsourcing to reference laboratories, both domestic and international, has emerged as the predominant bridging strategy, with external testing commonly conducted at academic hospital reference laboratories (40%) and commercial laboratories (51.4%). KHCC experience aligns with this international pattern, with outsourcing of testing distributed across medulloblastoma molecular subgrouping (NanoString nCounter gene expression profiling), pan-cancer RNA sequencing, fusion gene analysis, and FISH.

The rate of conclusive molecular results in our cohort varied considerably by tumor type, a finding consistent with the known technical challenges inherent to each assay and tumor subtype (18, 21). Medulloblastoma achieved the highest conclusive results at 94%, reflecting both the relative maturity of RNA-based subgrouping platforms and the generally adequate tissue yield from these tumors. By contrast, ependymoma had the lowest conclusive results at 33%, and embryonal tumors yielded conclusive results in only 63% of cases. These lower rates likely reflect a combination of insufficient tissue quantity, RNA degradation in FFPE samples, and the intrinsic molecular complexity of these rare entities (22).

In resource-limited settings, targeted case selection based on the likelihood of actionable findings may represent a rational, cost-effective strategy that maximizes clinical utility per dollar spent, rather than a limitation to be overcome. A previous study by Amayiri et al. reported on an initial cohort of 32 pediatric patients who underwent TruSight RNA pan-cancer sequencing between March 2022 and April 2023, with a median TAT of 23.5 days (range 15–49 days) at a cost of USD 1,000 per sample (23). The study expands on that work in several important ways: it encompasses a larger cohort of 105 patients over a broader time period (2021–2023), includes four distinct test types beyond TruSight, and captures both pediatric and adult patients. Notably, the median TAT in the current series was longer at 26 days (range 13–140 days), likely reflecting the inclusion of additional test types with varying processing requirements and a higher proportion of cases with logistical complications such as sample loss or re-shipment. The rate of actionable alterations identified in the prior series was notably higher (59%), which the authors attributed to selection bias toward diagnostically challenging cases and those with a high prior probability of harboring targetable mutations, a pattern consistent with the more selective case referral criteria applied in that initial experience. The broader cohort in the current study yielded an overall actionable target rate of 27%, rising to 65% in low-grade glioma. This lower rate, compared with the prior series, likely reflects differences in case selection. The optimal balance between comprehensive systematic testing and selective case-based outsourcing will depend on institutional resources, available in-house capacity, and the specific clinical context. Together, these two reports from KHCC provide a comprehensive picture of the evolution of molecular outsourcing from a selective feasibility exercise to an institutionally embedded diagnostic strategy.

The clinical value of molecular testing in this cohort must be understood in the context of its intended purpose for each tumor type, rather than assessed solely through the lens of diagnostic reclassification. In medulloblastoma, which comprised 52% of this cohort, molecular subgrouping was performed not to establish or change the diagnosis, but to guide risk stratification and treatment intensity. The most clinically impactful finding in our series was that molecular testing enabled targeted therapy. This expands therapeutic options for patients who would otherwise be managed with conventional chemotherapy alone. BRAF alterations, encompassing *KIAA1549::BRAF* fusions and BRAF p.V600E mutations, are the dominant oncogenic drivers in pediatric low-grade gliomas, occurring in most cases through MAPK pathway activation (24). Multiple targeted agents have demonstrated meaningful efficacy against these alterations, including the FDA-approved combination of dabrafenib and trametinib for *BRA*F V600E-mutant pediatric low-grade gliomas, and tovorafenib, a type II RAF inhibitor with FDA accelerated approval for *BRAF*-altered recurrent pediatric low-grade gliomas (25). The clinical significance of identifying *BRAF* p.V600E mutations in pediatric low-grade glioma is underscored by the landmark phase 2 randomized trial by Bouffet et al., which demonstrated clear superiority of dabrafenib plus trametinib over standard carboplatin-vincristine chemotherapy as first-line therapy (26) leading to FDA approval in this indication. These findings reiterate the findings from our previously published studies.

From a strategic standpoint, our data suggest important considerations for test selection in resource-limited settings. The TruSight pan-cancer RNA sequencing panel, despite its higher per-sample cost (USD 2,327 vs USD 704 for focused fusion analysis), demonstrated broad utility across multiple tumor types and achieved approximately 50% yield in detecting actionable alterations. This comprehensive approach is particularly valuable when the specific molecular alteration is uncertain, when multiple potential targets are being considered, or when the diagnosis itself requires molecular confirmation. In contrast, focused low-grade fusion gene analysis offers significant cost savings and may be appropriate when clinical, radiological, and histopathological features strongly suggest a *BRAF*-altered low-grade glioma and the primary question is simply confirming the presence of a *BRAF* fusion. However, a limitation of focused panels is that they may miss other actionable alterations or fail to resolve diagnostically ambiguous cases, potentially necessitating subsequent comprehensive testing, adding costs and delays. In our cohort, the limited utilization of the focused fusion panel (4 cases) compared to TruSight (48 cases) reflects our MDC’s tendency to favor comprehensive testing, recognizing that a single comprehensive test, while more expensive upfront, may be more cost-effective than sequential testing approaches. Centers developing outsourcing programs must weigh these considerations in light of their case mix, diagnostic confidence, available therapies, and financial constraints.

In research protocols and specialized centers, medulloblastoma subgroup assignment may inform treatment stratification, though subgroup-directed therapy is not universally standard of care and varies by institutional protocol and geographic region. Subgroup assignment provided prognostic information for all 55 conclusively tested medulloblastoma cases, though the extent to which this information directly altered treatment decisions in our setting was not systematically captured; none of which required a diagnostic change precisely because the diagnosis was already established and the molecular test was serving its intended stratification purpose (27). In contrast, for low-grade glioma, high-grade glioma, and non-MB embryonal tumors, molecular testing served a dual role: confirming or refining the diagnosis and identifying actionable therapeutic targets. The 5% overall diagnostic change rate, while numerically modest, is therefore an incomplete measure of impact; the true value of testing lies in the risk-adapted treatment decisions it enabled across all tumor types (23, 28).

The overall turnaround time of 26 days in our series is clinically relevant, particularly considering established benchmarks from high-income settings. Clinical decision-making for high-grade gliomas must occur within 2–3 weeks from initial surgery to ensure timely initiation of chemoradiation and potential enrollment in clinical trials, and it is therefore recommended that turnaround times be no longer than one week for *MGMT* promoter analysis and two weeks for NGS panels (19). In our opinion, this is one of the major limitations of a sustainable outsourcing program.

Specimen loss during international shipment and initial tissue inadequacy, which require resubmission, represent significant operational limitations of outsourcing models, directly contributing to delays in individual cases. These factors are not indicative of laboratory processing time but rather of the logistical vulnerabilities inherent in international sample transport.

The total cost of 108,986 JOD over two years represents a substantial institutional investment, with the TruSight pan-cancer RNAseq panel accounting for most expenditure at 79,296 JOD. Most often, this cost was covered through the same routes as the other diagnostic tests at KHCC, with only a few exceptions in which the patient/family bore the cost of the test. The costs of the tests are considered significant relative to the Jordanian GDP per capita, which is estimated at 4016.83 US dollars in 2024 (https://tradingeconomics.com/jordan/gdp-per-capita).

This study has several important limitations. First, the retrospective design limits our ability to prospectively control case selection or systematically capture all relevant clinical variables. Second, the overall molecular testing success rate of 92% (109/119 samples yielding definitive results) means that 8% of cases did not receive conclusive molecular diagnoses despite outsourcing, reflecting technical challenges inherent to FFPE-based molecular testing, particularly for small biopsies or RNA-degraded specimens. Third, there was a lack of systematic follow-up data regarding therapeutic implementation and clinical outcomes; while we identified actionable molecular alterations in 28 patients (27%), we did not systematically capture whether these patients received matched targeted therapies or whether clinical management changed based on molecular findings. Fourth, the absence of a comparator cohort, such as patients managed without molecular testing or those diagnosed before the outsourcing program was established, precludes direct assessment of the impact of molecular diagnostics on survival, progression-free survival, or other patient-centered outcomes. Fifth, case selection was determined by MDC review rather than systematic criteria, introducing potential selection bias toward cases with higher diagnostic uncertainty or greater likelihood of harboring actionable alterations. Finally, the costs and turnaround times reported reflect the specific KHCC-SickKids arrangement and may not generalize to other institutional partnerships or geographic contexts, particularly where shipping logistics, regulatory requirements, or laboratory pricing structures differ substantially. Future prospective studies should track not only molecular findings but also subsequent treatment choices and clinical outcomes to fully assess the real-world impact of molecular diagnostics in limited-resource settings.

### Moving forward

KHCC has since developed in-house capacity for select *BRAF* alteration testing, with current costs of 200 JOD (USD ~282) for *BRAF* mutation analysis by PCR, and 250 JOD (USD ~352) for *BRAF* fusion testing by FISH, compared to 500 JOD (USD ~704) per sample outsourced for the same analyses. This represents a cost reduction of approximately 50–60% for these specific tests, illustrating the long-term financial case for in-house capacity building. However, comprehensive panels such as the TruSight pan-cancer RNAseq, which identified most actionable alterations in this cohort, remains to be implemented. In addition, since April 2024, methylation profiling is done on all CNS tumors at KHCC and became the major modality for medulloblastoma molecular grouping and subgrouping.

## Recommendations

The long-term solution lies in establishing regional molecular diagnostic capacity, whether through in-house platform development, regional laboratory networks, or participation in structured twinning programs with internationally accredited centers (29). The MNP-Outreach Consortium, for example, was specifically established to facilitate global adoption of methylation-based CNS tumor classification in LMICs and represents the type of collaborative infrastructure that institutions such as KHCC should actively engage with (30).

## Conclusion

This study demonstrates that international outsourcing of molecular diagnostics for CNS tumors is a feasible and clinically valuable bridging strategy for centers in limited-resource settings. At KHCC, outsourced molecular testing of 109 samples from 105 patients enabled WHO CNS5-compliant integrated diagnoses and identified actionable therapeutic targets in a substantial proportion of patients, most notably those with low-grade glioma. These findings translated into meaningful opportunities to initiate molecularly guided therapies for patients who would otherwise have received only conventional treatment. Nevertheless, there are logistical, clinical, and financial limitations for this approach.

Our experience serves as a practical model in comparable LMIC settings with limited resources. Outsourcing enables centers to deliver contemporary, WHO-compliant oncology care while longer-term infrastructure is being developed. The path forward requires deliberate investment in regional molecular diagnostic capacity, through in-house platform development, regional laboratory networks, and structured twinning programs with internationally accredited centers, to achieve durable, equitable access to precision neuro-oncology for patients in resource-limited settings.

## Data Availability

The raw data supporting the conclusions of this article will be made available by the authors, without undue reservation.
